# Supplementary material for: Age-specific effects of weight-based body size on fracture risk in later life: a lifecourse Mendelian randomisation study
Source: Eur J Epidemiol. 2023 May 3;38(7):795–807. doi: 10.1007/s10654-023-00986-6 (PMC10276076; doi:10.1007/s10654-023-00986-6)
Supplement: Supplementary file 1 — Supplementary Material 1 [file 10654_2023_986_MOESM1_ESM.pdf]

Full title: Age-specific effects of weight-based body size on fracture risk in later life: A lifecourse Mendelian randomisation study

Running title: Weight-based body size and fracture risk

Grace M. Power, MSc.,[1*] Jon Tobias, PhD.,[1,2] Timothy M. Frayling, PhD.,[3] Jess Tyrrell, PhD.,[3] April Hartley, PhD.,[2] Jon Heron, PhD.,[1] George Davey Smith, FRS.,[1,4¶] Tom G. Richardson, PhD.,[1¶]

[1] *MRC Integrative Epidemiology Unit, University of Bristol, Bristol, UK.*

[2] *Musculoskeletal Research Unit, Translational Health Sciences, Bristol Medical School, University of Bristol, Bristol, UK.*

[3] *Genetics of Complex Traits, College of Medicine and Health, University of Exeter, Exeter, UK.*

[4] *NIHR Bristol Biomedical Research Centre Bristol, University Hospitals Bristol and Weston NHS Foundation Trust, University of Bristol, Bristol, UK.*

[¶] These authors contributed equally to this work.

[*] Corresponding author: Grace M Power - grace.power@bristol.ac.uk - +44 (0)117 331 0098

Population Health Sciences, Bristol Medical School, University of Bristol, Oakfield House, Oakfield Grove, Bristol, BS8 2BN, United Kingdom

S1 Table: A summary of the genetic datasets used in this study

Variable	Trait	Sample size	Year	First author	PubMed ID	Ancestry
Exposure	Childhood body size	453169	2020	Richardson TG	32376654	European
Exposure	Adult body size	453169	2020	Richardson TG	32376654	European
Exposure	Birthweight	261932	2021	NA	NA	European
Mediator	Vitamin D	449913	2021	NA	NA	European
Mediator	Calcium	432151	2021	NA	NA	European
Mediator	eBMD	278932	2021	NA	NA	European
Mediator	Bioavailable testosterone	414074	2021	NA	NA	European
Mediator	Total testosterone	364411	2021	NA	NA	European
Mediator	SHBG	428137	2021	NA	NA	European
Outcome	Fracture risk in later life	416795	2018	Morris JA	30598549	European
Exposure and outcome (Sensitivity analysis)	Fat mass	453957	2021	NA	NA	European
Exposure and outcome (Sensitivity analysis)	Fat-free mass	454669	2021	NA	NA	European

S2 Table: Input for web application to calculate expected bias and Type 1 error rate under the null for genetically proxied childhood body size and fracture risk in later life

Field	Input
Outcome is:	Binary
Coefficient of determination (R2) of risk factor on genetic variants:	0.6
Sample size for exposure:	49109
Sample size for outcome:	413809
Number of instruments:	313
Bias of the observational estimate (OLS estimate):	0.8-0.9
[For binary outcome only] Proportion of cases (if 1:1 ratio of cases to controls, then proportion is 0.5):	0.106

S3 Table: Output from web application to calculate expected bias and Type 1 error rate under the null for genetically proxied childhood body size and fracture risk in later life

Using specified value of concentration parameter			Using conservative value of concentration parameter		
Overlap proportion	Bias	Type 1 error rate	Overlap proportion	Bias	Type 1 error rate
0.0	0.000	0.05	0.0	0.000	0.05
0.1	0.000	0.05	0.1	0.000	0.05
0.2	0.001	0.05	0.2	0.001	0.05
0.3	0.001	0.05	0.3	0.001	0.05
0.4	0.001	0.06	0.4	0.001	0.06
0.5	0.002	0.06	0.5	0.002	0.06
0.6	0.002	0.06	0.6	0.002	0.06
0.7	0.002	0.07	0.7	0.002	0.07
0.8	0.003	0.07	0.8	0.003	0.07
0.9	0.003	0.08	0.9	0.003	0.08
1.0	0.003	0.08	1.0	0.003	0.08

S4 Table: Two-step Mendelian randomisation analyses for childhood and adult body size onto potential mediators and potential mediators onto fracture risk in later life and estimated bone mineral density

Exposure*	Mediator/Outcome	nSNPs**	Beta	Standard Error	P value	Odds Ratio	Lower CI***	Upper CI***	Mendelian Randomization (MR)	Method	Sex
Childhood body size	eBMD	313	0.26	0.03	3E-16	--	0.20	0.33	Univariable MR	Inverse variance weighted	All
Childhood body size	eBMD	313	0.25	0.03	7E-21	--	0.20	0.30	Univariable MR	Weighted median	All
Childhood body size	eBMD	313	0.36	0.07	1E-06	--	0.21	0.50	Univariable MR	MR Egger	All
Childhood body size	eBMD	142	0.18	0.04	7E-06	--	0.10	0.26	Univariable MR	Inverse variance weighted	Female
Childhood body size	eBMD	142	0.22	0.04	5E-10	--	0.15	0.29	Univariable MR	Weighted median	Female
Childhood body size	eBMD	142	0.27	0.09	4E-03	--	0.09	0.44	Univariable MR	MR Egger	Female
Childhood body size	eBMD	69	0.36	0.06	3E-08	--	0.23	0.49	Univariable MR	Inverse variance weighted	Male
Childhood body size	eBMD	69	0.37	0.05	6E-13	--	0.27	0.47	Univariable MR	Weighted median	Male
Childhood body size	eBMD	69	0.44	0.15	4E-03	--	0.15	0.73	Univariable MR	MR Egger	Male
Adult body size	eBMD	580	0.20	0.02	1E-17	--	0.15	0.25	Univariable MR	Inverse variance weighted	All
Adult body size	eBMD	580	0.22	0.02	1E-29	--	0.18	0.26	Univariable MR	Weighted median	All
Adult body size	eBMD	580	0.24	0.07	3E-04	--	0.11	0.37	Univariable MR	MR Egger	All
Adult body size	eBMD	221	0.16	0.04	9E-06	--	0.09	0.24	Univariable MR	Inverse variance weighted	Female
Adult body size	eBMD	221	0.16	0.03	3E-07	--	0.10	0.23	Univariable MR	Weighted median	Female
Adult body size	eBMD	221	0.22	0.11	5E-02	--	0.00	0.44	Univariable MR	MR Egger	Female
Adult body size	eBMD	168	0.34	0.04	4E-14	--	0.25	0.42	Univariable MR	Inverse variance weighted	Male
Adult body size	eBMD	168	0.37	0.05	7E-16	--	0.28	0.45	Univariable MR	Weighted median	Male
Adult body size	eBMD	168	0.52	0.13	2E-04	--	0.26	0.78	Univariable MR	MR Egger	Male
Childhood body size	Calcium	313	-0.10	0.02	6E-07	--	-0.15	-0.06	Univariable MR	Inverse variance weighted	All
Childhood body size	Calcium	313	-0.09	0.02	1E-05	--	-0.13	-0.05	Univariable MR	Weighted median	All
Childhood body size	Calcium	313	-0.11	0.05	2E-02	--	-0.20	-0.02	Univariable MR	MR Egger	All
Childhood body size	Vitamin D	313	-0.07	0.02	5E-05	--	-0.10	-0.04	Univariable MR	Inverse variance weighted	All
Childhood body size	Vitamin D	313	-0.06	0.02	2E-03	--	-0.10	-0.02	Univariable MR	Weighted median	All
Childhood body size	Vitamin D	313	-0.11	0.04	3E-03	--	-0.19	-0.04	Univariable MR	MR Egger	All
Adult body size	Calcium	580	-0.05	0.02	6E-03	--	-0.08	-0.01	Univariable MR	Inverse variance weighted	All
Adult body size	Calcium	580	-0.07	0.02	6E-05	--	-0.10	-0.03	Univariable MR	Weighted median	All
Adult body size	Calcium	580	-0.09	0.05	7E-02	--	-0.20	0.01	Univariable MR	MR Egger	All

Adult body size	Vitamin D	580	-0.18	0.01	1E-37	--	-0.21	-0.15	Univariable MR	Inverse variance weighted	All
Adult body size	Vitamin D	580	-0.19	0.02	3E-32	--	-0.22	-0.16	Univariable MR	Weighted median	All
Adult body size	Vitamin D	580	-0.18	0.04	2E-05	--	-0.26	-0.10	Univariable MR	MR Egger	All
Childhood body size	eBMD	267	0.20	0.04	3E-07	--	0.12	0.27	Multivariable MR	Inverse variance weighted	All
Adult body size	eBMD	535	0.09	0.03	8E-03	--	0.02	0.15	Multivariable MR	Inverse variance weighted	All
Childhood body size	eBMD	132	0.21	0.04	5E-07	--	0.13	0.29	Multivariable MR	Inverse variance weighted	Female
Adult body size	eBMD	202	0.04	0.04	3E-01	--	-0.03	0.12	Multivariable MR	Inverse variance weighted	Female
Childhood body size	eBMD	58	0.20	0.07	8E-03	--	0.05	0.35	Multivariable MR	Inverse variance weighted	Male
Adult body size	eBMD	161	0.14	0.06	3E-02	--	0.02	0.27	Multivariable MR	Inverse variance weighted	Male
Childhood body size	Calcium	267	-0.11	0.03	1E-04	--	-0.17	-0.05	Multivariable MR	Inverse variance weighted	All
Adult body size	Calcium	535	0.01	0.03	6E-01	--	-0.04	0.06	Multivariable MR	Inverse variance weighted	All
Childhood body size	Vitamin D	267	0.07	0.02	3E-03	--	0.02	0.11	Multivariable MR	Inverse variance weighted	All
Adult body size	Vitamin D	535	-0.22	0.02	4E-30	--	-0.26	-0.18	Multivariable MR	Inverse variance weighted	All
eBMD	Fractures	548	-0.48	0.02	5E-150	0.62	0.60	0.64	Univariable MR	Inverse variance weighted	All
eBMD	Fractures	548	-0.48	0.02	3E-91	0.62	0.59	0.65	Univariable MR	Weighted median	All
eBMD	Fractures	548	-0.54	0.04	9E-41	0.58	0.54	0.63	Univariable MR	MR Egger	All
eBMD	Fractures	323	-0.51	0.02	2E-112	0.60	0.57	0.63	Univariable MR	Inverse variance weighted	Female
eBMD	Fractures	323	-0.56	0.03	9E-77	0.57	0.54	0.61	Univariable MR	Weighted median	Female
eBMD	Fractures	323	-0.59	0.05	1E-29	0.55	0.51	0.61	Univariable MR	MR Egger	Female
eBMD	Fractures	206	-0.37	0.03	5E-40	0.69	0.65	0.73	Univariable MR	Inverse variance weighted	Male
eBMD	Fractures	206	-0.47	0.04	1E-38	0.63	0.58	0.67	Univariable MR	Weighted median	Male
eBMD	Fractures	206	-0.47	0.06	2E-13	0.62	0.55	0.70	Univariable MR	MR Egger	Male
Calcium	Fractures	355	-0.01	0.02	8E-01	0.99	0.95	1.04	Univariable MR	Inverse variance weighted	All
Calcium	Fractures	355	0.01	0.03	7E-01	1.01	0.95	1.08	Univariable MR	Weighted median	All
Calcium	Fractures	355	0.01	0.05	8E-01	1.01	0.92	1.10	Univariable MR	MR Egger	All
Vitamin D	Fractures	131	0.02	0.03	5E-01	1.02	0.96	1.09	Univariable MR	Inverse variance weighted	All
Vitamin D	Fractures	131	-0.02	0.05	8E-01	0.98	0.89	1.09	Univariable MR	Weighted median	All
Vitamin D	Fractures	131	0.00	0.05	1E+00	1.00	0.91	1.10	Univariable MR	MR Egger	All
Calcium	eBMD	355	-0.04	0.02	4E-02		-0.07	0.00	Univariable MR	Inverse variance weighted	All
Calcium	eBMD	355	-0.04	0.02	2E-02		-0.06	-0.01	Univariable MR	Weighted median	All
Calcium	eBMD	355	-0.02	0.03	5E-01		-0.09	0.05	Univariable MR	MR Egger	All
Vitamin D	eBMD	131	-0.01	0.02	6E-01		-0.06	0.03	Univariable MR	Inverse variance weighted	All

Vitamin D	eBMD	131	-0.04	0.02	1E-02		-0.07	-0.01	Univariable MR	Weighted median	All
Vitamin D	eBMD	131	-0.02	0.03	5E-01		-0.09	0.05	Univariable MR	MR Egger	All

* Results represent an increase in the exposure variable

** Number of single-nucleotide polymorphisms (SNPs)

*** 95% Confidence Interval

S5 Table: Two-step Mendelian randomisation analyses for childhood and adult body size onto sex-specific mediators and mediators onto fracture risk in later life and estimated bone mineral density

Exposure*	Mediator/Outcome	nSNPs **	Beta	Standard Error	P value	Odds Ratio	Lower CI***	Upper CI***	Mendelian Randomization (MR)	Method	Sex
Childhood body size	Bioavailable testosterone	142	0.10	0.03	1E-03	--	0.04	0.16	Univariable MR	Inverse variance weighted	Female
Childhood body size	Bioavailable testosterone	142	0.09	0.03	5E-03	--	0.03	0.15	Univariable MR	Weighted median	Female
Childhood body size	Bioavailable testosterone	142	0.15	0.07	4E-02	--	0.01	0.29	Univariable MR	MR Egger	Female
Childhood body size	Bioavailable testosterone	69	-0.28	0.04	7E-13	--	-0.35	-0.20	Univariable MR	Inverse variance weighted	Male
Childhood body size	Bioavailable testosterone	69	-0.24	0.04	9E-11	--	-0.31	-0.16	Univariable MR	Weighted median	Male
Childhood body size	Bioavailable testosterone	69	-0.25	0.09	6E-03	--	-0.42	-0.08	Univariable MR	MR Egger	Male
Childhood body size	Total testosterone	142	0.01	0.01	3E-01	--	-0.01	0.04	Univariable MR	Inverse variance weighted	Female
Childhood body size	Total testosterone	142	0.02	0.02	4E-01	--	-0.02	0.05	Univariable MR	Weighted median	Female
Childhood body size	Total testosterone	142	-0.01	0.03	8E-01	--	-0.07	0.05	Univariable MR	MR Egger	Female
Childhood body size	Total testosterone	69	-1.21	0.21	2E-08	--	-1.62	-0.79	Univariable MR	Inverse variance weighted	Male
Childhood body size	Total testosterone	69	-1.19	0.15	1E-14	--	-1.49	-0.89	Univariable MR	Weighted median	Male
Childhood body size	Total testosterone	69	-0.86	0.48	8E-02	--	-0.95	-0.78	Univariable MR	MR Egger	Male
Childhood body size	SHBG	142	-0.16	0.04	3E-04	--	-0.25	-0.07	Univariable MR	Inverse variance weighted	Female
Childhood body size	SHBG	142	-0.11	0.03	7E-04	--	-0.17	-0.05	Univariable MR	Weighted median	Female
Childhood body size	SHBG	142	-0.28	0.10	7E-03	--	-0.48	-0.08	Univariable MR	MR Egger	Female
Childhood body size	SHBG	69	-0.18	0.07	2E-02	--	-0.32	-0.03	Univariable MR	Inverse variance weighted	Male
Childhood body size	SHBG	69	-0.13	0.04	2E-03	--	-0.20	-0.05	Univariable MR	Weighted median	Male
Childhood body size	SHBG	69	-0.10	0.17	6E-01	--	-0.42	0.23	Univariable MR	MR Egger	Male
Adult body size	Bioavailable testosterone	221	0.41	0.03	5E-45	--	0.35	0.47	Univariable MR	Inverse variance weighted	Female
Adult body size	Bioavailable testosterone	221	0.35	0.03	2E-32	--	0.29	0.41	Univariable MR	Weighted median	Female
Adult body size	Bioavailable testosterone	221	0.16	0.09	7E-02	--	-0.01	0.33	Univariable MR	MR Egger	Female
Adult body size	Bioavailable testosterone	168	-0.22	0.03	8E-13	--	-0.27	-0.16	Univariable MR	Inverse variance weighted	Male
Adult body size	Bioavailable testosterone	168	-0.24	0.03	1E-14	--	-0.30	-0.18	Univariable MR	Weighted median	Male
Adult body size	Bioavailable testosterone	168	-0.35	0.09	2E-04	--	-0.53	-0.17	Univariable MR	MR Egger	Male
Adult body size	Total testosterone	221	0.08	0.02	4E-06	--	0.04	0.11	Univariable MR	Inverse variance weighted	Female
Adult body size	Total testosterone	221	0.05	0.02	1E-02	--	0.01	0.08	Univariable MR	Weighted median	Female
Adult body size	Total testosterone	221	0.03	0.05	6E-01	--	-0.07	0.13	Univariable MR	MR Egger	Female

Adult body size	Total testosterone	168	-1.66	0.11	2E-51	--	-1.87	-1.44	Univariable MR	Inverse variance weighted	Male
Adult body size	Total testosterone	168	-1.56	0.12	2E-38	--	-1.80	-1.33	Univariable MR	Weighted median	Male
Adult body size	Total testosterone	168	-1.37	0.33	6E-05	--	-2.02	-0.72	Univariable MR	MR Egger	Male
Adult body size	SHBG	221	-0.56	0.04	5E-54	--	-0.64	-0.49	Univariable MR	Inverse variance weighted	Female
Adult body size	SHBG	221	-0.51	0.03	6E-67	--	-0.57	-0.45	Univariable MR	Weighted median	Female
Adult body size	SHBG	221	-0.27	0.11	1E-02	--	-0.49	0.06	Univariable MR	MR Egger	Female
Adult body size	SHBG	168	-0.42	0.04	3E-29	--	-0.49	-0.34	Univariable MR	Inverse variance weighted	Male
Adult body size	SHBG	168	-0.38	0.03	3E-31	--	-0.45	-0.32	Univariable MR	Weighted median	Male
Adult body size	SHBG	168	-0.18	0.11	1E-01	--	-0.40	0.04	Univariable MR	MR Egger	Male
Childhood body size	Bioavailable testosterone	132	-0.20	0.04	2E-06	--	-0.28	-0.12	Multivariable MR	Inverse variance weighted	Female
Adult body size	Bioavailable testosterone	202	0.51	0.04	7E-38	--	0.43	0.59	Multivariable MR	Inverse variance weighted	Female
Childhood body size	Bioavailable testosterone	58	-0.18	0.05	8E-04	--	-0.28	-0.07	Multivariable MR	Inverse variance weighted	Male
Adult body size	Bioavailable testosterone	161	-0.11	0.04	1E-02	--	-0.20	-0.02	Multivariable MR	Inverse variance weighted	Male
Childhood body size	Total testosterone	132	-0.04	0.02	6E-02	--	-0.08	0.00	Multivariable MR	Inverse variance weighted	Female
Adult body size	Total testosterone	202	0.09	0.02	2E-06	--	0.06	0.13	Multivariable MR	Inverse variance weighted	Female
Childhood body size	Total testosterone	58	0.01	0.23	1E+00	--	-0.45	0.47	Multivariable MR	Inverse variance weighted	Male
Adult body size	Total testosterone	161	-1.68	0.20	3E-17	--	-2.07	-1.29	Multivariable MR	Inverse variance weighted	Male
Childhood body size	SHBG	132	0.25	0.06	1E-05	--	0.14	0.35	Multivariable MR	Inverse variance weighted	Female
Adult body size	SHBG	202	-0.69	0.05	4E-40	--	-0.79	-0.59	Multivariable MR	Inverse variance weighted	Female
Childhood body size	SHBG	58	0.21	0.08	1E-02	--	0.05	0.37	Multivariable MR	Inverse variance weighted	Male
Adult body size	SHBG	161	-0.55	0.07	4E-15	--	-0.69	-0.41	Multivariable MR	Inverse variance weighted	Male
Bioavailable testosterone	Fracture risk in later life	143	-0.10	0.03	1E-03	0.91	0.86	0.96	Univariable MR	Inverse variance weighted	Female
Bioavailable testosterone	Fracture risk in later life	143	-0.10	0.05	2E-02	0.90	0.82	0.98	Univariable MR	Weighted median	Female
Bioavailable testosterone	Fracture risk in later life	143	-0.16	0.06	9E-03	0.85	0.75	0.96	Univariable MR	MR Egger	Female
Bioavailable testosterone	Fracture risk in later life	101	-0.16	0.04	7E-05	0.85	0.79	0.92	Univariable MR	Inverse variance weighted	Male
Bioavailable testosterone	Fracture risk in later life	101	-0.21	0.06	6E-04	0.81	0.72	0.91	Univariable MR	Weighted median	Male
Bioavailable testosterone	Fracture risk in later life	101	-0.19	0.07	9E-03	0.83	0.72	0.95	Univariable MR	MR Egger	Male
Total testosterone	Fracture risk in later life	92	-0.15	0.07	3E-02	0.86	0.76	0.98	Univariable MR	Inverse variance weighted	Female
Total testosterone	Fracture risk in later life	92	-0.33	0.10	1E-03	0.72	0.59	0.88	Univariable MR	Weighted median	Female
Total testosterone	Fracture risk in later life	92	-0.31	0.12	9E-03	0.74	0.59	0.92	Univariable MR	MR Egger	Female
Total testosterone	Fracture risk in later life	186	-0.02	0.01	4E-02	0.98	0.97	1.00	Univariable MR	Inverse variance weighted	Male
Total testosterone	Fracture risk in later life	186	-0.01	0.01	3E-01	0.99	0.97	1.00	Univariable MR	Weighted median	Male

Total testosterone	Fracture risk in later life	186	-0.02	0.01	2E-01	0.98	0.95	1.01	Univariable MR	MR Egger	Male
SHBG	Fracture risk in later life	261	0.09	0.02	1E-04	1.10	1.05	1.15	Univariable MR	Inverse variance weighted	Female
SHBG	Fracture risk in later life	261	0.08	0.03	2E-02	1.08	1.01	1.16	Univariable MR	Weighted median	Female
SHBG	Fracture risk in later life	261	0.02	0.04	7E-01	1.02	0.93	1.10	Univariable MR	MR Egger	Female
SHBG	Fracture risk in later life	300	0.01	0.02	6E-01	1.01	0.97	1.06	Univariable MR	Inverse variance weighted	Male
SHBG	Fracture risk in later life	300	0.02	0.04	7E-01	1.02	0.94	1.10	Univariable MR	Weighted median	Male
SHBG	Fracture risk in later life	300	-0.01	0.03	9E-01	0.99	0.93	1.06	Univariable MR	MR Egger	Male
Bioavailable testosterone	eBMD	143	0.17	0.02	3E-12	--	0.12	0.21	Univariable MR	Inverse variance weighted	Female
Bioavailable testosterone	eBMD	143	0.12	0.02	2E-08	--	0.08	0.16	Univariable MR	Weighted median	Female
Bioavailable testosterone	eBMD	143	0.18	0.05	2E-04	--	0.09	0.28	Univariable MR	MR Egger	Female
Bioavailable testosterone	eBMD	101	0.20	0.03	2E-13	--	0.15	0.25	Univariable MR	Inverse variance weighted	Male
Bioavailable testosterone	eBMD	101	0.24	0.03	3E-18	--	0.19	0.29	Univariable MR	Weighted median	Male
Bioavailable testosterone	eBMD	101	0.22	0.05	2E-05	--	0.12	0.31	Univariable MR	MR Egger	Male
Total testosterone	eBMD	92	0.17	0.05	2E-04	--	0.08	0.26	Univariable MR	Inverse variance weighted	Female
Total testosterone	eBMD	92	0.11	0.04	2E-02	--	0.02	0.19	Univariable MR	Weighted median	Female
Total testosterone	eBMD	92	0.18	0.08	2E-02	--	0.03	0.34	Univariable MR	MR Egger	Female
Total testosterone	eBMD	186	0.01	0.01	1E-01	--	0.00	0.02	Univariable MR	Inverse variance weighted	Male
Total testosterone	eBMD	186	0.01	0.01	2E-02	--	0.00	0.02	Univariable MR	Weighted median	Male
Total testosterone	eBMD	186	0.02	0.01	7E-02	--	0.00	0.04	Univariable MR	MR Egger	Male
SHBG	eBMD	261	-0.14	0.02	2E-17	--	-0.18	-0.11	Univariable MR	Inverse variance weighted	Female
SHBG	eBMD	261	-0.09	0.02	2E-09	--	-0.12	-0.06	Univariable MR	Weighted median	Female
SHBG	eBMD	261	-0.10	0.03	2E-03	--	-0.15	-0.04	Univariable MR	MR Egger	Female
SHBG	eBMD	300	-0.05	0.01	4E-04	--	-0.08	-0.02	Univariable MR	Inverse variance weighted	Male
SHBG	eBMD	300	-0.03	0.02	3E-02	--	-0.06	0.00	Univariable MR	Weighted median	Male
SHBG	eBMD	300	-0.02	0.02	4E-01	--	-0.06	0.02	Univariable MR	MR Egger	Male

* Results represent an increase in the exposure variable

** Number of single-nucleotide polymorphisms (SNPs)

*** 95% Confidence Interval

S6 Table: Univariable and multivariable Mendelian randomisation analyses for birthweight, childhood and adult body size onto fracture risk in later life and estimated bone mineral density

Exposure*	Outcome	nSNPs**	Beta	Standard Error	P value	Odds ratio (95% confidence interval)	Mendelian Randomization (MR)
Birthweight	Fracture risk in later life	174	0.08	0.04	3E-02	1.08 (1.01, 1.16)	Univariable MR
Childhood body size	Fracture risk in later life	313	-0.12	0.04	5E-03	0.89 (0.82, 0.96)	Univariable MR
Adult body size	Fracture risk in later life	580	0.08	0.03	2E-02	1.08 (1.01, 1.16)	Univariable MR
Birthweight	eBMD	174	-0.08	0.03	1E-02	(-0.14, 0.02)	Univariable MR
Childhood body size	eBMD	313	0.26	0.03	3E-16	(0.20, 0.33)	Univariable MR
Adult body size	eBMD	580	0.2	0.02	1E-17	(0.15, 0.25)	Univariable MR
Birthweight	Fracture risk in later life	148	0.1	0.03	3E-03	1.10 (1.03, 1.17)	Multivariable MR
Childhood body size	Fracture risk in later life	203	-0.25	0.06	7E-05	0.78 (0.69, 0.88)	Multivariable MR
Adult body size	Fracture risk in later life	396	0.19	0.05	3E-04	1.21 (1.09, 1.35)	Multivariable MR
Birthweight	eBMD	148	-0.11	0.02	1E-05	(-0.16, -0.06)	Multivariable MR
Childhood body size	eBMD	203	0.21	0.05	2E-05	(0.11, 0.30)	Multivariable MR
Adult body size	eBMD	396	0.11	0.04	1E-02	(0.02, 0.19)	Multivariable MR

* Results represent an increase in the exposure variable

** Number of single-nucleotide polymorphisms (SNPs)

S7 Table: Univariable and multivariable mendelian randomisation analyses for childhood and adult body size onto fat free mass index and fat mass index in adulthood

Exposure*	Outcome	nSNPs **	Beta	Standard Error	P value	Lower CI***	Upper CI***	Mendelian Randomization (MR)	Method	Sex
Childhood body size	Fat free mass index	313	0.74	0.02	3E-195	0.69	0.79	Univariable MR	Inverse variance weighted	All
Childhood body size	Fat free mass index	313	0.61	0.02	9E-191	0.57	0.65	Univariable MR	Weighted Median	All
Childhood body size	Fat free mass index	313	0.82	0.06	1E-37	0.71	0.93	Univariable MR	MR-Egger	All
Adult body size	Fat free mass index	580	1.00	0.01	1E-300	0.98	1.02	Univariable MR	Inverse variance weighted	All
Adult body size	Fat free mass index	580	0.95	0.01	1E-300	0.93	0.98	Univariable MR	Weighted Median	All
Adult body size	Fat free mass index	580	1.09	0.03	1E-167	1.04	1.14	Univariable MR	MR-Egger	All
Childhood body size	Fat free mass index	267	0.18	0.02	8E-23	0.14	0.21	Multivariable MR	Inverse variance weighted	All
Adult body size	Fat free mass index	535	0.91	0.02	1E-300	0.88	0.94	Multivariable MR	Inverse variance weighted	All
Childhood body size	Fat mass index	313	0.78	0.03	7E-154	0.72	0.83	Univariable MR	Inverse variance weighted	All
Childhood body size	Fat mass index	313	0.75	0.02	2E-219	0.70	0.80	Univariable MR	Weighted Median	All
Childhood body size	Fat mass index	313	0.99	0.06	1E-39	0.86	1.12	Univariable MR	MR-Egger	All
Adult body size	Fat mass index	580	1.30	0.01	1E-300	1.29	1.32	Univariable MR	Inverse variance weighted	All
Adult body size	Fat mass index	580	1.27	0.02	1E-300	1.24	1.31	Univariable MR	Weighted Median	All
Adult body size	Fat mass index	580	1.30	0.02	2E-248	1.26	1.35	Univariable MR	MR-Egger	All
Childhood body size	Fat mass index	267	-0.05	0.01	5E-04	-0.08	-0.02	Multivariable MR	Inverse variance weighted	All
Adult body size	Fat mass index	535	1.33	0.01	1E-300	1.30	1.35	Multivariable MR	Inverse variance weighted	All
Childhood body size	Fat free mass index	142	0.63	0.03	2E-90	0.57	0.69	Univariable MR	Inverse variance weighted	Female
Childhood body size	Fat free mass index	142	0.52	0.02	6E-119	0.48	0.56	Univariable MR	Weighted Median	Female
Childhood body size	Fat free mass index	142	0.78	0.07	5E-21	0.64	0.91	Univariable MR	MR-Egger	Female

Adult body size	Fat free mass index	221	0.84	0.02	1E-300	0.81	0.88	Univariable MR	Inverse variance weighted	Female
Adult body size	Fat free mass index	221	0.75	0.02	1E-300	0.71	0.78	Univariable MR	Weighted Median	Female
Adult body size	Fat free mass index	221	1.06	0.05	2E-54	0.96	1.16	Univariable MR	MR-Egger	Female
Childhood body size	Fat free mass index	132	0.19	0.02	2E-15	0.14	0.23	Multivariable MR	Inverse variance weighted	Female
Adult body size	Fat free mass index	202	0.75	0.02	4E-255	0.71	0.79	Multivariable MR	Inverse variance weighted	Female
Childhood body size	Fat mass index	142	0.72	0.03	1E-101	0.65	0.78	Univariable MR	Inverse variance weighted	Female
Childhood body size	Fat mass index	142	0.63	0.03	3E-123	0.58	0.69	Univariable MR	Weighted Median	Female
Childhood body size	Fat mass index	142	0.96	0.07	2E-26	0.82	1.10	Univariable MR	MR-Egger	Female
Adult body size	Fat mass index	221	1.11	0.01	1E-300	1.08	1.14	Univariable MR	Inverse variance weighted	Female
Adult body size	Fat mass index	221	1.07	0.02	1E-300	1.02	1.11	Univariable MR	Weighted Median	Female
Adult body size	Fat mass index	221	1.22	0.05	9E-72	1.13	1.31	Univariable MR	MR-Egger	Female
Childhood body size	Fat mass index	132	0.08	0.02	2E-04	0.04	0.12	Multivariable MR	Inverse variance weighted	Female
Adult body size	Fat mass index	202	1.06	0.02	1E-300	1.03	1.10	Multivariable MR	Inverse variance weighted	Female
Childhood body size	Fat free mass index	69	0.81	0.04	4E-98	0.73	0.88	Univariable MR	Inverse variance weighted	Male
Childhood body size	Fat free mass index	69	0.66	0.03	1E-82	0.59	0.72	Univariable MR	Weighted Median	Male
Childhood body size	Fat free mass index	69	0.94	0.09	1E-16	0.77	1.11	Univariable MR	MR-Egger	Male
Adult body size	Fat free mass index	168	0.94	0.02	1E-300	0.89	0.98	Univariable MR	Inverse variance weighted	Male
Adult body size	Fat free mass index	168	0.83	0.02	1E-300	0.79	0.87	Univariable MR	Weighted Median	Male
Adult body size	Fat free mass index	168	1.16	0.06	6E-43	1.04	1.28	Univariable MR	MR-Egger	Male
Childhood body size	Fat free mass index	58	0.28	0.03	1E-17	0.21	0.34	Multivariable MR	Inverse variance weighted	Male
Adult body size	Fat free mass index	161	0.77	0.03	2E-171	0.71	0.82	Multivariable MR	Inverse variance weighted	Male

Childhood body size	Fat mass index	69	0.91	0.05	6E-72	0.81	1.01	Univariable MR	Inverse variance weighted	Male
Childhood body size	Fat mass index	69	0.81	0.04	2E-80	0.73	0.89	Univariable MR	Weighted Median	Male
Childhood body size	Fat mass index	69	1.10	0.11	2E-14	0.88	1.32	Univariable MR	MR-Egger	Male
Adult body size	Fat mass index	168	1.18	0.02	1E-300	1.14	1.22	Univariable MR	Inverse variance weighted	Male
Adult body size	Fat mass index	168	1.11	0.03	1E-300	1.06	1.17	Univariable MR	Weighted Median	Male
Adult body size	Fat mass index	168	1.26	0.06	4E-46	1.14	1.38	Univariable MR	MR-Egger	Male
Childhood body size	Fat mass index	58	0.12	0.04	9E-04	0.05	0.19	Multivariable MR	Inverse variance weighted	Male
Adult body size	Fat mass index	161	1.10	0.03	1E-276	1.04	1.17	Multivariable MR	Inverse variance weighted	Male

* Results represent an increase in the exposure variable

** Number of single-nucleotide polymorphisms (SNPs)

*** 95% Confidence Interval

S8 Table: Univariable and multivariable Mendelian randomisation analyses for childhood and adult body size onto fracture risk in later life, accounting for fat free mass index and fat mass index

Exposure*	Accounting for	nSNPs **	Beta	Standard Error	P value	Odds Ratio	Lower CI***	Upper CI***	Mendelian Randomization (MR)	Method	Sex
Childhood body size	Fat free mass index	267	-0.20	0.06	8E-04	0.82	0.73	0.92	Multivariable MR	Inverse variance weighted	All
Adult body size	Fat free mass index	374	0.13	0.05	8E-03	1.14	1.04	1.25	Multivariable MR	Inverse variance weighted	All
Childhood body size	Fat mass index	267	-0.27	0.05	3E-07	0.76	0.68	0.84	Multivariable MR	Inverse variance weighted	All
Adult body size	Fat mass index	407	0.19	0.04	1E-07	1.20	1.12	1.29	Multivariable MR	Inverse variance weighted	All

* Results represent an increase in the exposure variable

** Number of single-nucleotide polymorphisms (SNPs)

*** 95% Confidence Interval

S9 Table: Univariable and multivariable Mendelian randomisation analyses for childhood and adult body size onto sex (female) to estimate sex-differential participation bias

Exposure*	nSNPs**	Beta	Standard Error	P value	Odds Ratio	Lower CI***	Upper CI***	Mendelian Randomization (MR)	Method
Childhood body size	298	-0.06	0.03	3E-02	0.94	0.89	0.99	Univariable MR	Inverse variance weighted
Childhood body size	298	-0.10	0.04	2E-02	0.90	0.83	0.98	Univariable MR	Weighted median
Childhood body size	298	-0.10	0.06	1E-01	0.91	0.80	1.03	Univariable MR	MR Egger
Adult body size	562	-0.05	0.02	5E-02	0.96	0.91	1.00	Univariable MR	Inverse variance weighted
Adult body size	562	-0.06	0.03	9E-02	0.99	0.89	1.01	Univariable MR	Weighted median
Adult body size	562	-0.09	0.07	2E-01	0.98	0.80	1.04	Univariable MR	MR Egger
Childhood body size	256	-0.02	0.04	6E-01	0.98	0.91	1.06	Multivariable MR	Inverse variance weighted
Adult body size	520	-0.03	0.03	3E-01	0.97	0.91	1.03	Multivariable MR	Inverse variance weighted

* Results represent an increase in the exposure variable

** Number of single-nucleotide polymorphisms (SNPs)

*** 95% Confidence Interval

S10 Table: Family-based univariable and multivariable Mendelian randomisation analyses for childhood and adult body size onto fracture risk in later life and estimated bone mineral density

Model	Exposure*	Outcome	P value	Odds Ratio	Lower CI**	Upper CI**	Clustered SE	Mendelian Randomization (MR)	Sample size
Between family	Childhood body size	Fracture risk in later life	9E-01	1.00	1.00	1.00	0.00	Univariable MR	39507
Within family	Childhood body size	Fracture risk in later life	6E-01	1.00	1.00	1.01	0.00	Univariable MR	39507
Between family	Childhood body size	eBMD	5E-05	1.03	1.02	1.05	0.01	Univariable MR	39507
Within family	Childhood body size	eBMD	5E-05	1.05	1.02	1.07	0.01	Univariable MR	39507
Between family	Adult body size	Fracture risk in later life	5E-01	1.00	1.00	1.00	0.00	Univariable MR	39507
Within family	Adult body size	Fracture risk in later life	2E-01	1.00	1.00	1.01	0.00	Univariable MR	39507
Between family	Adult body size	eBMD	1E-02	1.02	1.01	1.04	0.01	Univariable MR	39507
Within family	Adult body size	eBMD	2E-05	1.05	1.02	1.07	0.01	Univariable MR	39507
Between family	Childhood body size	Fracture risk in later life	7E-01	1.00	1.00	1.00	0.00	Multivariable MR	39507
Within family	Childhood body size	Fracture risk in later life	1E+00	1.00	0.99	1.01	0.00	Multivariable MR	39507
Between family	Childhood body size	eBMD	8E-04	1.03	1.01	1.04	0.01	Multivariable MR	39507
Within family	Childhood body size	eBMD	7E-03	1.03	1.01	1.06	0.01	Multivariable MR	39507

* Results represent an increase in the exposure variable

** 95% Confidence Interval
